# Footling Breech Delivery in an Emergency Department Without Obstetrical Services: A Case Report

**DOI:** 10.7759/cureus.58604

**Published:** 2024-04-19

**Authors:** Kara Bragg, Bradley Bragg

**Affiliations:** 1 Emergency Medicine, Mayo Clinic, Jacksonville, USA

**Keywords:** loveset maneuver, burns-marshall method, mauriceau maneuver, pinard maneuver, case report, breech delivery, precipitous delivery, emergency delivery, footling breech

## Abstract

Hospitals without formal obstetric services place the emergency physician in the position of managing potentially complicated precipitous labor and delivery such as breech presentations. Breech deliveries pose an increased risk of significant morbidity and mortality to both the mother and fetus. Recent emphasis on cesarean section as the optimal delivery method for breech presentation has decreased education and comfort levels with breech vaginal deliveries. This case study highlights a patient who presented to a suburban emergency department (ED) in active labor with a footling breech presentation. No obstetrical services were available. The delivery was successful, and the neonate was resuscitated. Though emergency medicine residents receive training in standard vaginal deliveries, most residents do not receive firsthand experience with difficult deliveries. This case highlights the need for policy and procedure and annual competency training for physicians and allied staff in ED facilities without obstetrical services. Structured protocols and annual simulation training will improve outcomes for imminent deliveries with potential complications.

## Introduction

The incidence of emergency department (ED) deliveries is not well reported [1]. Malpresentations during delivery increase the risk of complications for both mother and fetus and can produce significant stress for the emergency physician [2]. Breech presentations typically occur in 3% to 4% of all deliveries [1]. This increases the potential for significant morbidity and mortality to both the mother and fetus [1]. Since the Term Breech Trial publication in 2004, the cesarean section has been the safest means of delivery for breech presentations [3]. Physicians now counsel women and recommend a cesarean section for the increased safety of both mother and baby in the event of a breech delivery [4]. This has consequently led to fewer vaginal breech deliveries and reduced instruction for this type of delivery [3,5]. With the emphasis on cesarean section, safe and effective methods, and care pathways have been poorly established in the obstetrical arena, making it even more challenging for the ED physician who rarely encounters deliveries, especially precipitous labor and high-risk cases [3]. Physicians who are in ED settings without formal obstetrical services, and where a cesarean section may not be feasible, may face an increased risk of adverse outcomes in patients with a complicated breech presentation [1]. While emergency medicine residencies typically have a competency requirement of ten spontaneous vaginal deliveries during training, it is unclear if residents are provided training in performing breech deliveries [5]. A recent survey showed that many program directors were not confident in their graduate’s abilities to manage shoulder dystocia and breech deliveries [5]. There can be substantial time after graduation from residency before an ED physician encounters a difficult delivery. Furthermore, the new evidence-based practice dictates the need for updated training and simulation to ensure providers have up-to-date training [3].

## Case presentation

A 22-year-old woman presented to our ED with diffuse abdominal pain at 4:40 am. She stated that the pain started at 11:30 pm the evening before and denied vaginal bleeding, vaginal discharge, urinary symptoms, fever, or other complaints. She denied knowledge of the pregnancy to the physician, however, she informed the triage nurse at intake that pregnancy was a possibility. She reported having a menstrual cycle three months before her presentation in the ED with no concerning features. She was presumed nulliparous and had no other significant medical history other than obesity. She was in no acute distress, and not obviously pregnant on initial examination. Abdominal examination revealed a protuberant, distended abdomen with diffuse tenderness and a mass suspected to be a fundal height 5cm above the umbilicus. A pelvic examination was quickly performed before the results of laboratory tests, and this revealed a foot protruding from the vaginal introitus. The patient’s gestational age was unknown given unreliable menstrual history. There were no formal obstetrical services available at the hospital and the patient presented on the night shift when gynecology staff were offsite. In the setting of an imminent footling breech delivery, the obstetrician/gynecologist on-call was paged and protocols for precipitous delivery previously arranged at our institution were initiated.

While the on-call obstetrician/gynecologist was en route to the hospital, two registered nurses including a former nurse midwife from the United Kingdom and a nurse with previous obstetrical experience reported to the emergency department to assist in the delivery of the infant. An obstetric delivery kit, neonatal resuscitative equipment, and a neonatal warmer were prepared. It was immediately apparent that the footling breech delivery was inevitable and that safe transport of the patient to another facility would not be a viable option. The patient was placed in McRoberts positioning, the physician used the Pinard procedure for delivery of the second leg and performed a mediolateral episiotomy. The physician executed the Lovset maneuver for delivery of the shoulders and reduction of a nuchal cord. The delivery was complicated by head entrapment requiring the Mauriceau-Smellie-Veit maneuver for successful extraction of the fetus.

The infant failed to respond adequately to initial stimulation and suctioning and required endotracheal intubation. Apgar scores were three at one minute, three at five minutes, and six at 10 minutes. The infant showed a dramatic improvement in color, activity, and spontaneous respirations following endotracheal intubation and assisted ventilation. The obstetrician arrived after delivery and intubation of the neonate to repair the episiotomy incision.

The infant and mother were transferred to a nearby hospital with a neonatal intensive care unit and postpartum support via ambulance. The complete blood count and a complete metabolic panel of the mother resulted during delivery without any concerning findings. The infant and mother were transferred before any blood typing and Rh factor labs. Urine and imaging were not obtained before the precipitous delivery. All remaining tests were deferred after delivery to the receiving facility at their request. The mother and child recovered fully without complication. The mother and child were lost to follow-up after discharge from the receiving facility.

## Discussion

Precipitous deliveries are defined as three hours or less from the onset of labor to delivery, however, all ED deliveries should be managed as precipitous deliveries [6]. All deliveries outside the hospital or in the ED require specialized equipment, training, and protocols [7]. ED and prehospital deliveries are associated with a high degree of complications for both mother and infant including cervical spine injury and asphyxiation for the baby, large perineal lacerations, retained placenta, and postpartum hemorrhage [6,8]. This is particularly pertinent for EDs without obstetrical facilities available within the same hospital.

**Figure 1:**
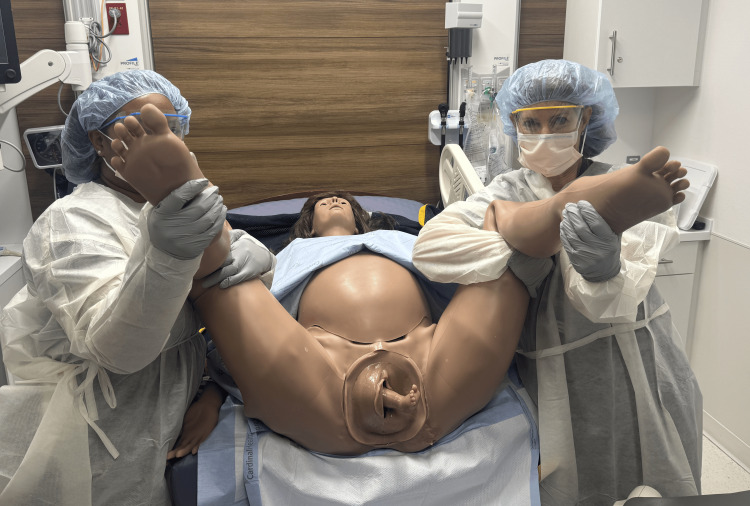
McRoberts position used when delivering in the dorsal lithotomy position to assist in opening pelvic outlet. Photographs: Taken at Mayo Clinic using the Victoria S2200 Advanced Obstetric Patient Simulator by Gaumard.

Breech deliveries account for 3% to 4% of deliveries [1]. Footling breech presentations are higher-risk vaginal deliveries [9]. If cesarean section is not an option due to staff resources, lack of surgical experience, or necessary facility capabilities, a series of maneuvers are indicated to provide for a successful vaginal delivery [1,6]. Place patient in the McRoberts position if in dorsal lithotomy birthing position which allows for the pelvic tilt facilitating delivery around the symphysis pubis (Figure 1) [10].

**Figure 2:**
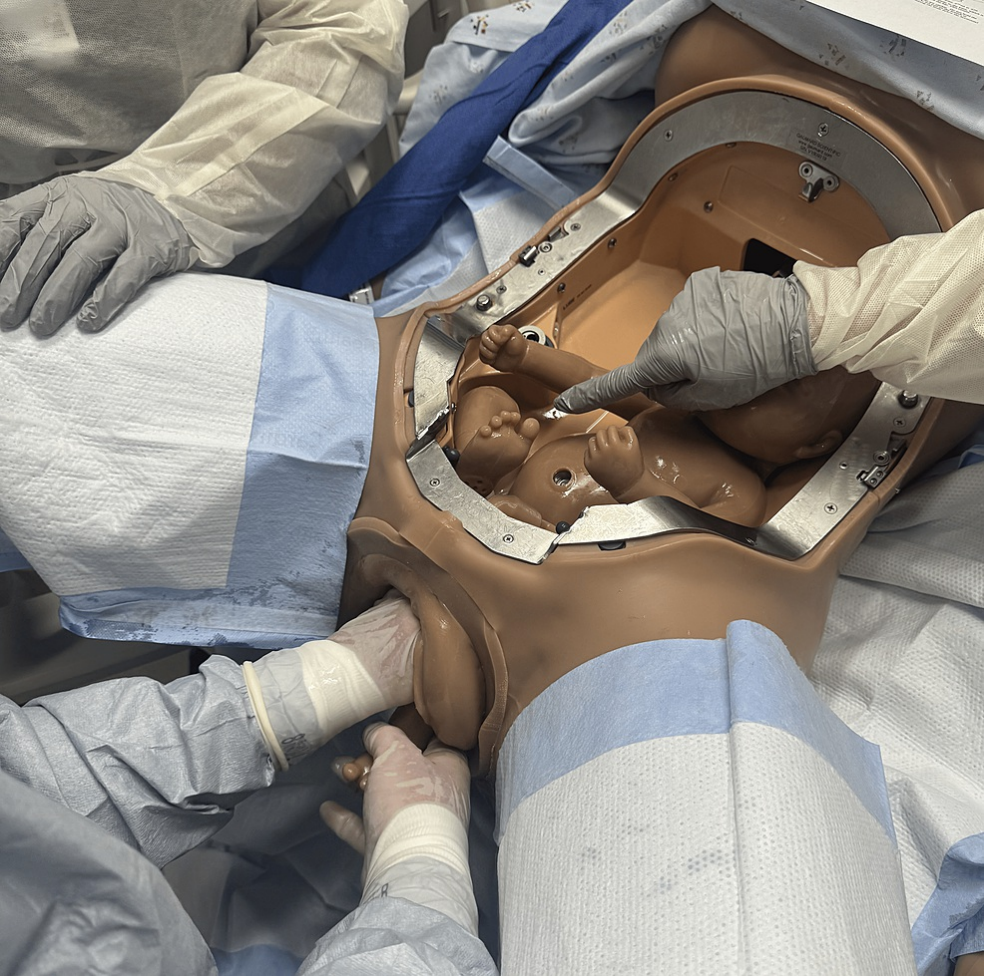
Pinard maneuver showing the intrauterine position of the leg and the area the physician will grasp with two fingers. Photographs: Taken at Mayo Clinic using the Victoria S2200 Advanced Obstetric Patient Simulator by Gaumard.

**Figure 3:**
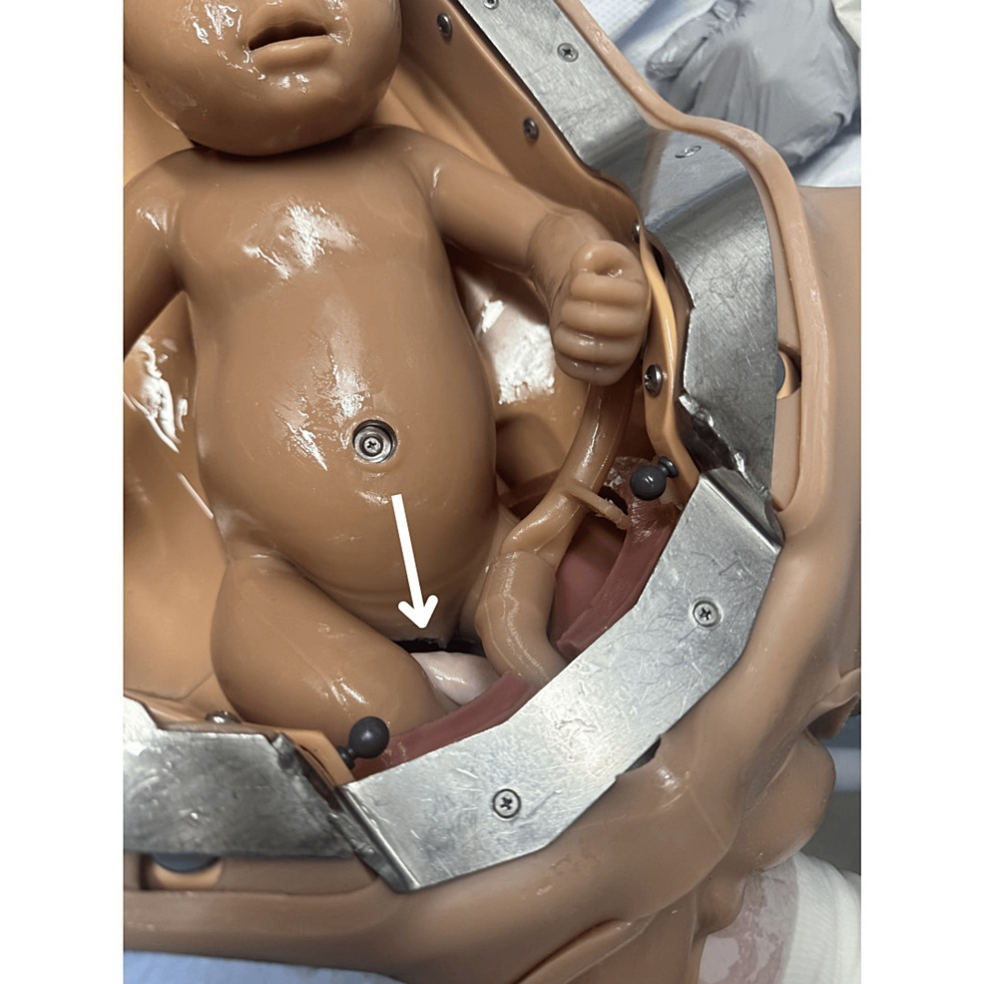
Pinard maneuver showing intrauterine view of the physician's fingers grasping the leg. Photographs: Taken at Mayo Clinic using the Victoria S2200 Advanced Obstetric Patient Simulator by Gaumard.

**Figure 4:**
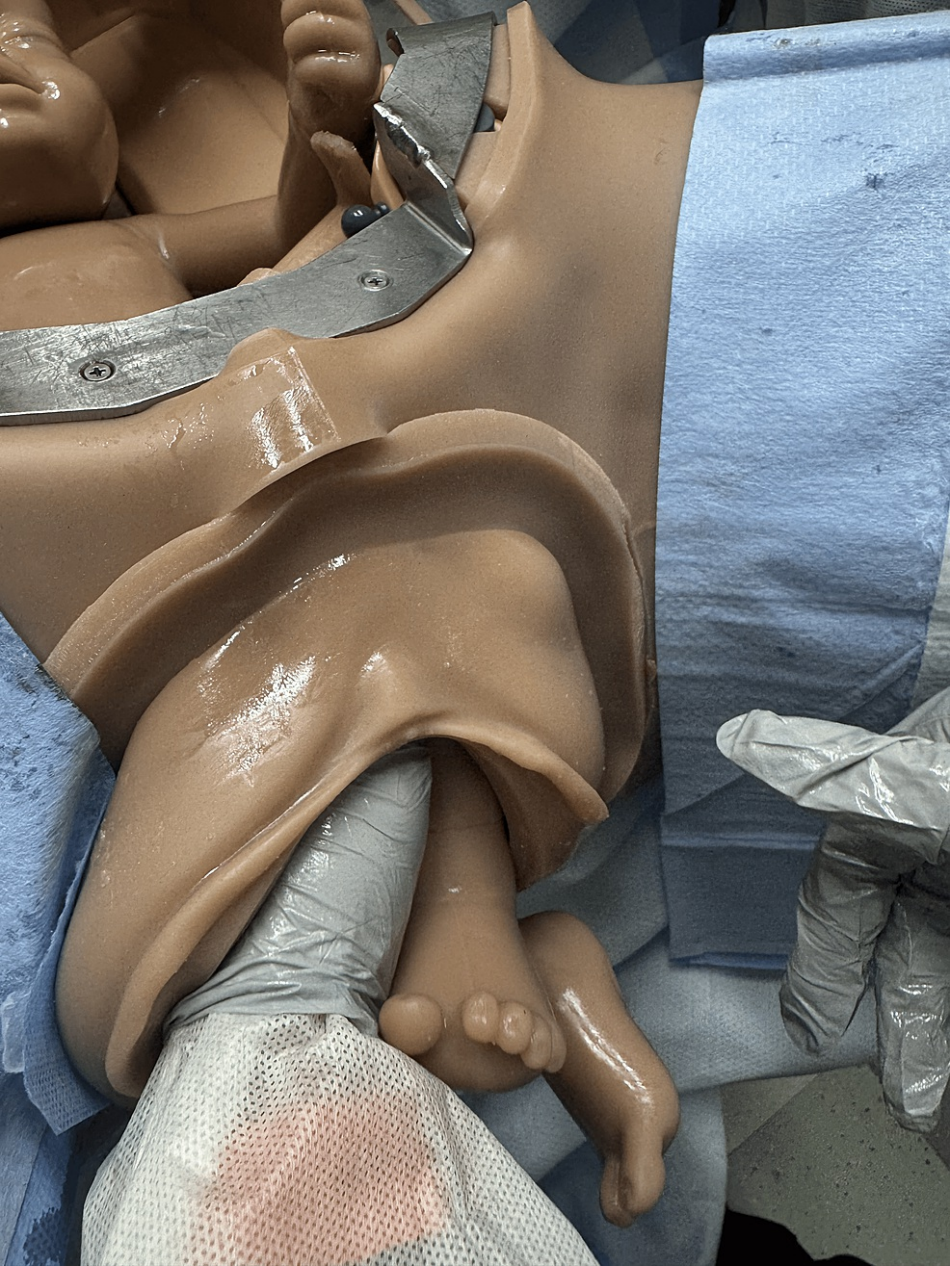
Pinard maneuver with completed delivery of both legs. Photographs: Taken at Mayo Clinic using the Victoria S2200 Advanced Obstetric Patient Simulator by Gaumard.

There is also evidence for facilitated birth with the mother positioned on hands and knees, though this may be limited by staffing comfort and location [11]. Initiate the delivery with the Pinard maneuver delivering both lower extremities by inserting two fingers into the vagina and “hooking” the leg medially to laterally just above the knee [12]. Flex the leg at the hip and abduct the extremity delivering the foot (Figures 2-4) [1,12].

**Figure 5:**
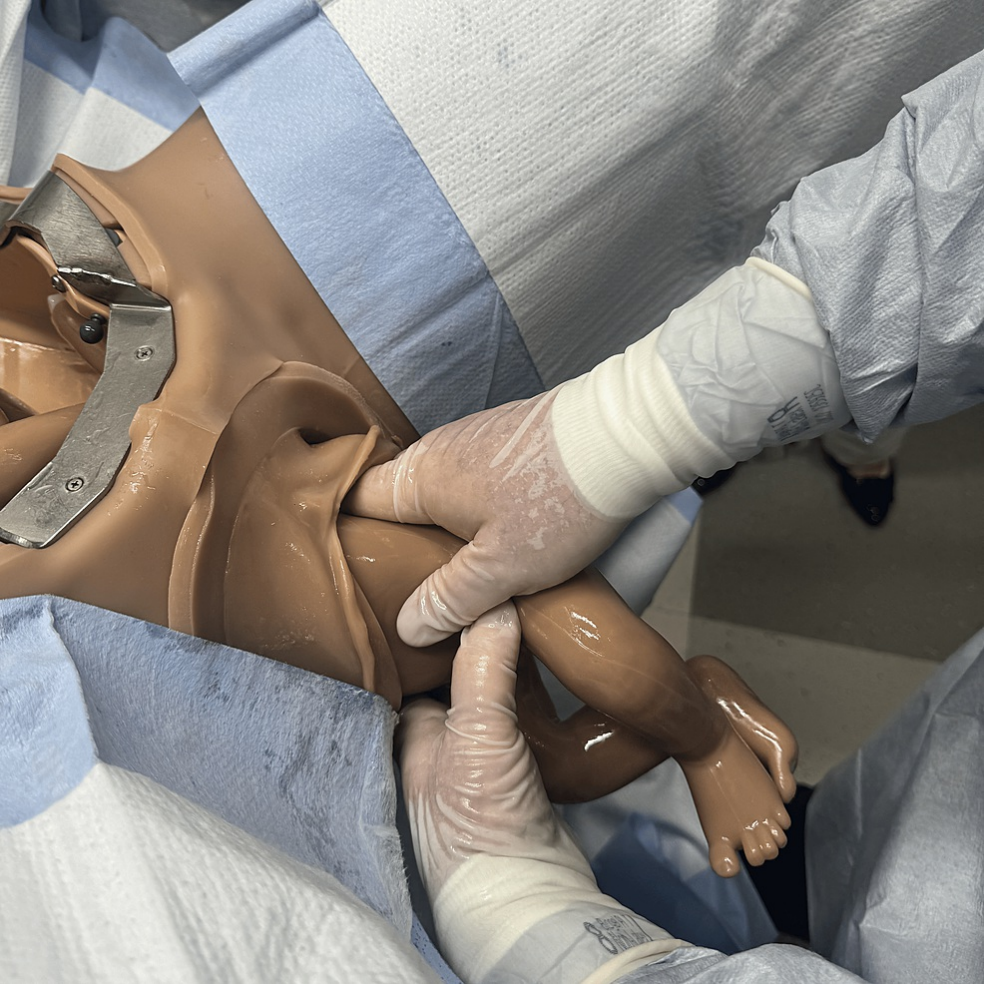
Loveset maneuver with grasping the baby's pelvis and rotating baby for delivery of shoulders. Photographs: Taken at Mayo Clinic using the Victoria S2200 Advanced Obstetric Patient Simulator by Gaumard.

**Figure 6:**
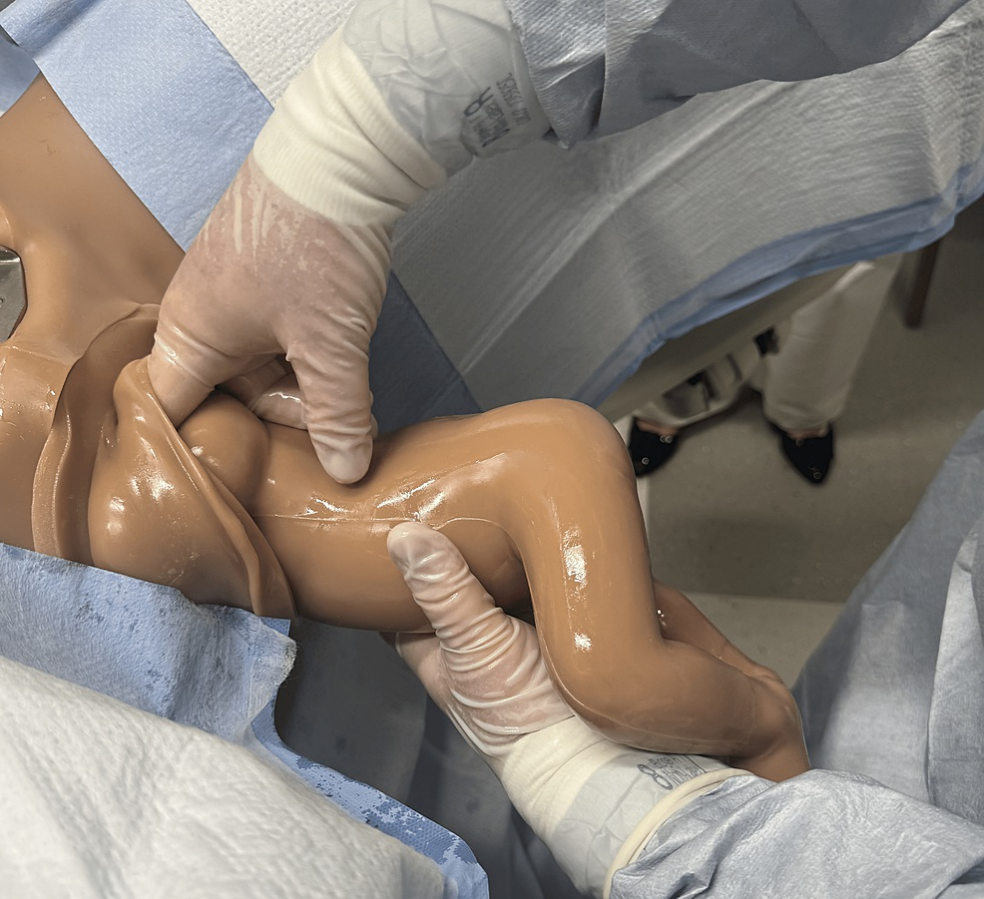
Loveset maneuver with finger sweep for delivery of the first shoulder and arm. Photographs: Taken at Mayo Clinic using the Victoria S2200 Advanced Obstetric Patient Simulator by Gaumard.

**Figure 7:**
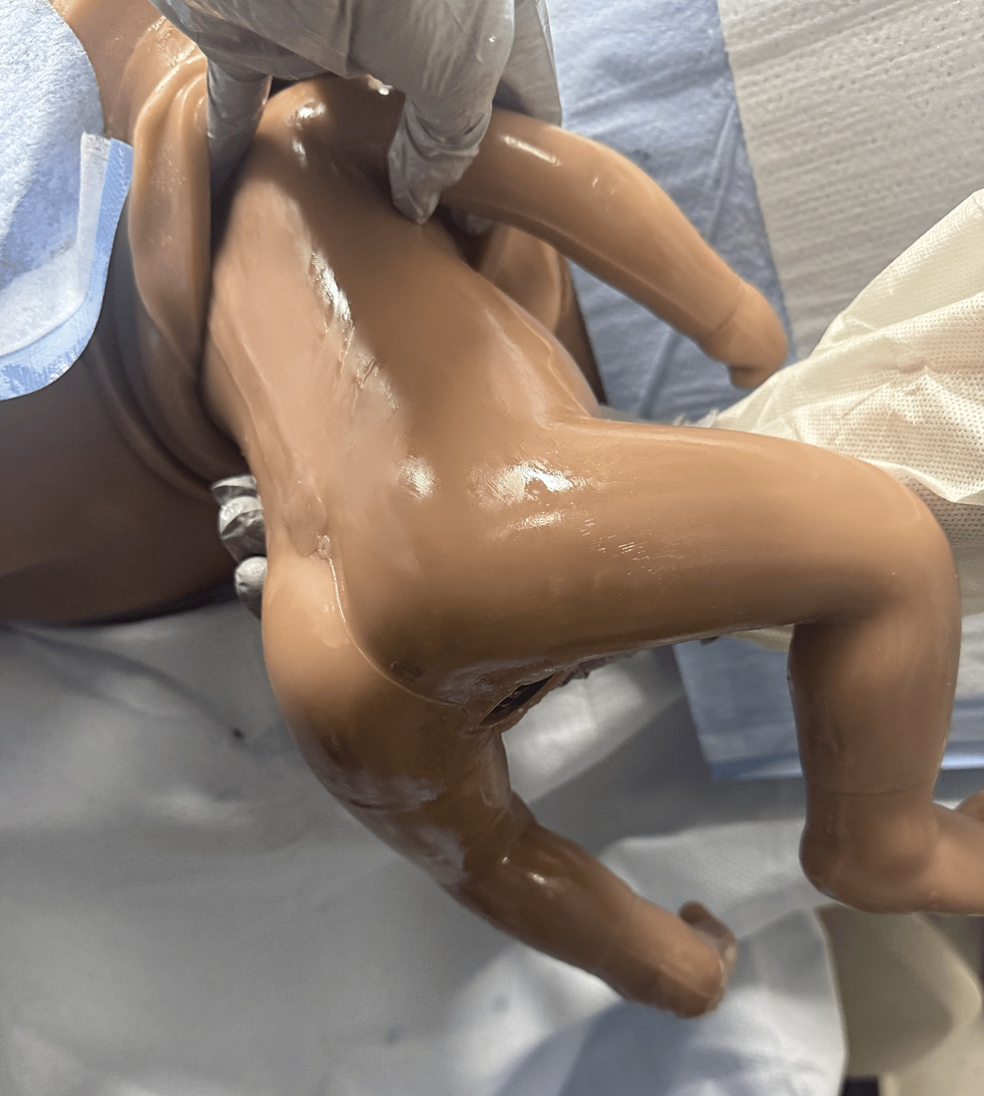
Loveset maneuver with positioning to deliver the second shoulder. Photographs: Taken at Mayo Clinic using the Victoria S2200 Advanced Obstetric Patient Simulator by Gaumard.

Upon delivery of the scapula, initiate the Lovset maneuver where the baby is maneuvered sideways to deliver one arm, then 180 degrees the other way to deliver the other arm [1,12]. Sweep arms over the chest of the fetus if either arm fails to present to assist in their delivery (Figures 5-7) [2].

**Figure 8:**
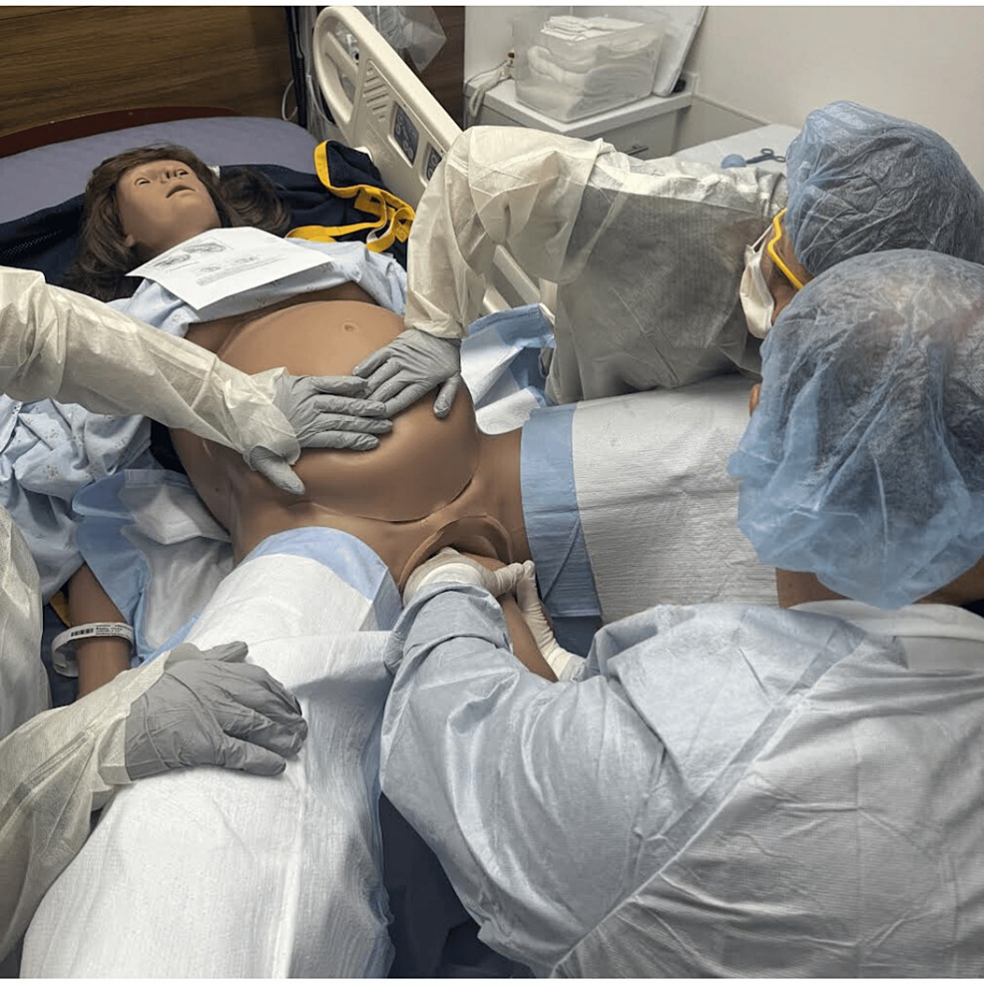
Mauriceau–Smellie–Veit maneuver showing pressure over the fundus of the uterus to assist in delivery of the head. Photographs: Taken at Mayo Clinic using the Victoria S2200 Advanced Obstetric Patient Simulator by Gaumard.

**Figure 9:**
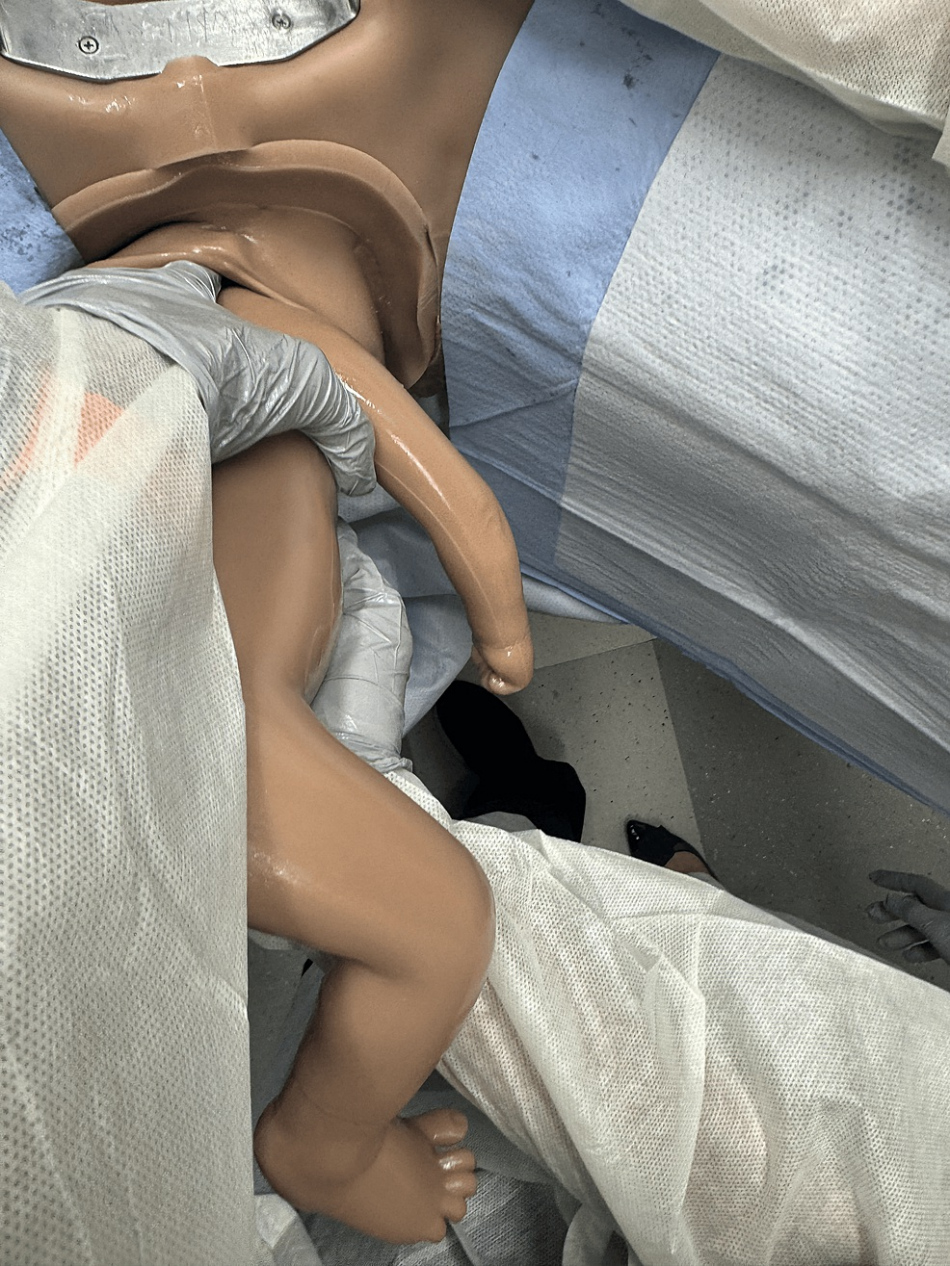
Mauriceau–Smellie–Veit maneuver showing one hand grasping the chin and flexing the head toward the baby's chest while the other hand simultaneously grasps along the occiput to guide the head in delivery. Photographs: Taken at Mayo Clinic using the Victoria S2200 Advanced Obstetric Patient Simulator by Gaumard.

The head can be delivered using one of two techniques. The Mauriceau maneuver as the physician inserts hands into the vaginal canal and applies pressure to the maxillary area [1]. This is followed by the application of strong suprapubic pressure to flex the baby’s head, avoiding any hyperextension of the neck risking spinal cord injury (Figures 8, 9) [2].

**Figure 10:**
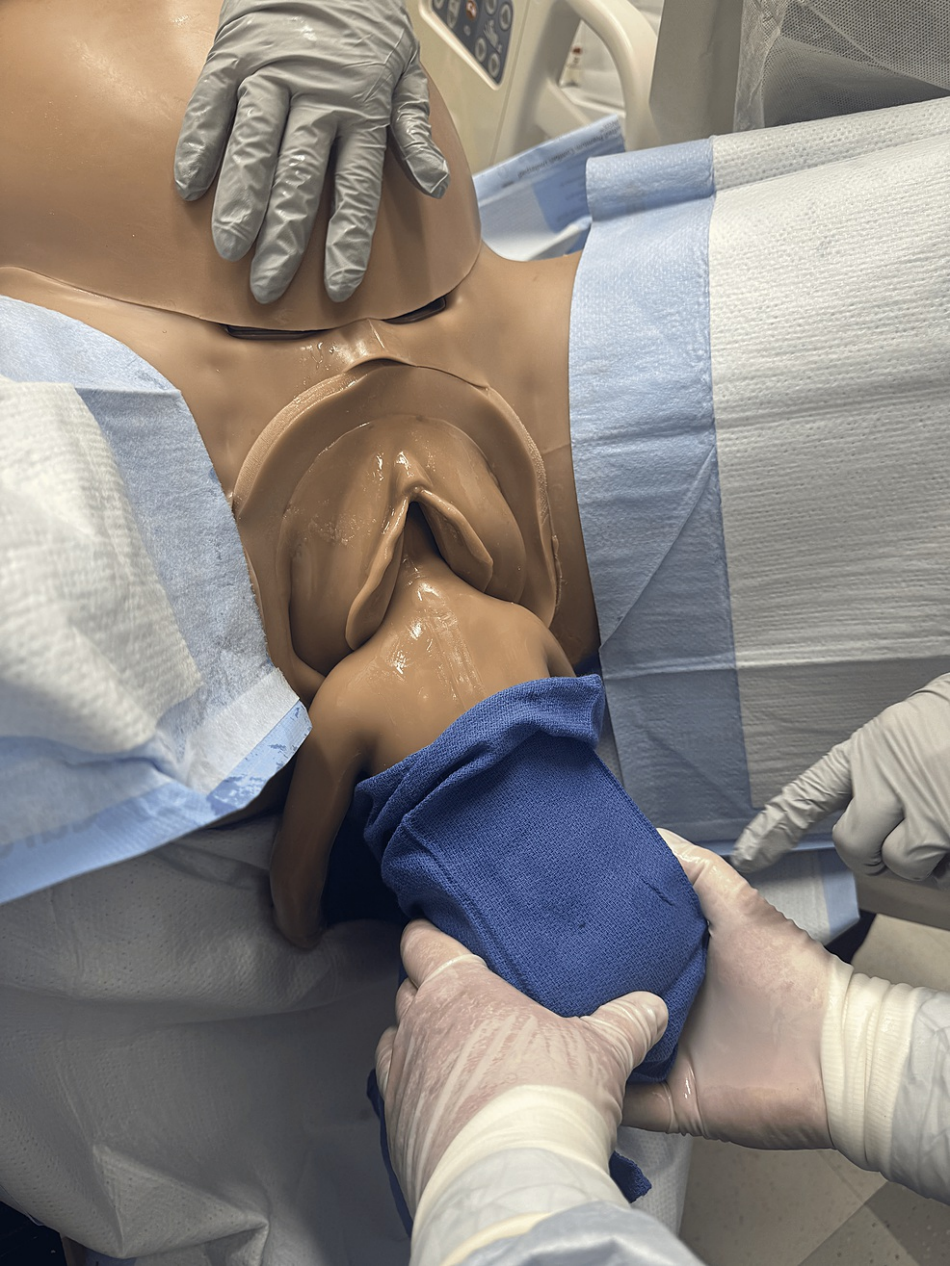
Burns–Marshall method showing baby "hanging" to allow gravity to assist in dilating cervix. The physician wraps baby in towel for warming. Photographs: Taken at Mayo Clinic using the Victoria S2200 Advanced Obstetric Patient Simulator by Gaumard.

**Figure 11:**
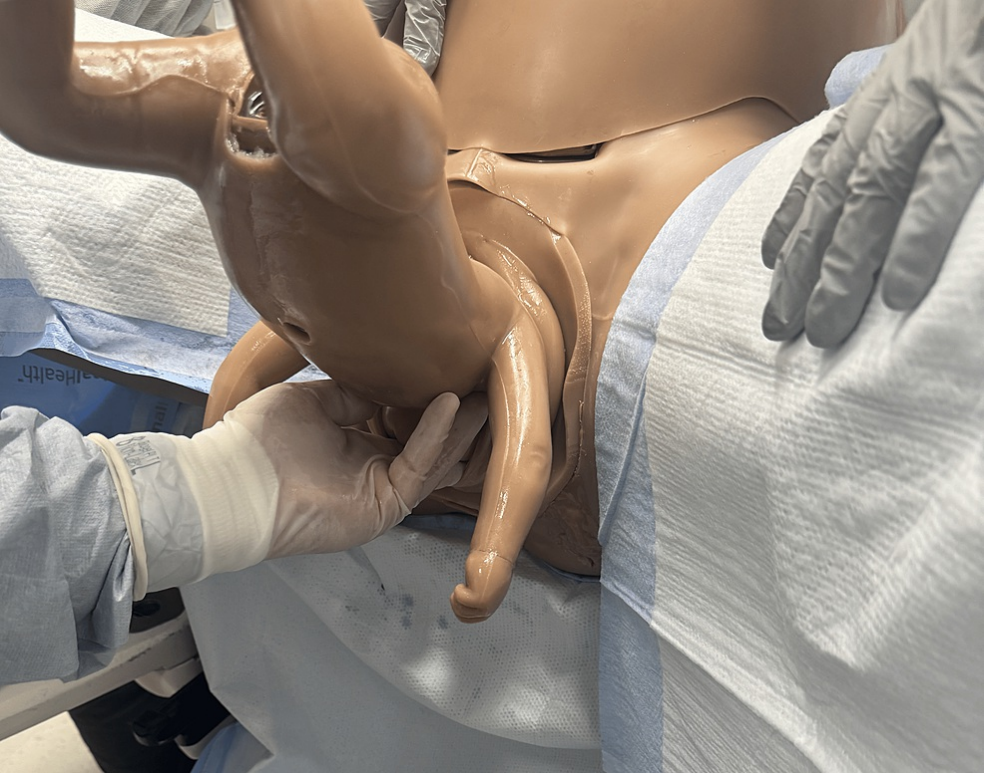
Burns–Marshall method showing physician grasping baby's chin guiding the delivery of the head. Photographs: Taken at Mayo Clinic using the Victoria S2200 Advanced Obstetric Patient Simulator by Gaumard.

**Figure 12:**
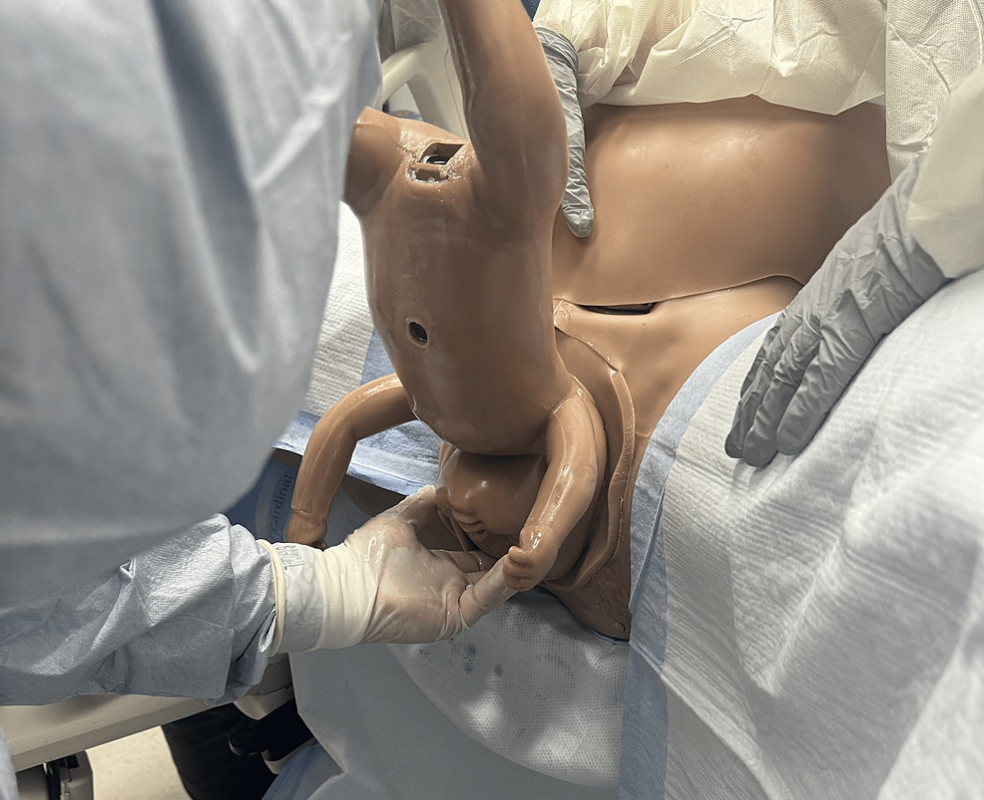
Burns–Marshall method showing final sweeping motion where physician holds baby's feet and delivers the baby. Photographs: Taken at Mayo Clinic using the Victoria S2200 Advanced Obstetric Patient Simulator by Gaumard.

A second method is a Burns-Marshall technique using a more “hands-off” approach in some instances to allow the fetal presenting part to dilate the cervix [1,6]. Aggressive traction increases the risk of cephalic entrapment which increases the risk for asphyxiation [2]. The baby is allowed to “hang” keeping the baby warm with a blanket and protecting the umbilical cord. At times, the baby may need help with positioning and new research shows assistance may be needed to guide the baby into position [11]. Once the nape of the neck is visible, the baby is delivered by holding the baby’s feet, easing the chin out by grasping with two fingers, and performing a sweeping motion to deliver once the chin has presented (Figures 10-12) [12].

The physician should simultaneously prepare for neonatal resuscitation during the delivery process. Staff should bring a neonatal warmer and the neonatal delivery kit with Kelly clamp, scissors, and a blanket to dry and stimulate the infant after birth [6]. Newer guidelines caution against aggressive suctioning indicating that suctioning is warranted only if airway obstruction is evident [1]. Do not perform endotracheal intubation or suctioning for meconium-stained amniotic fluid unless performed for airway obstruction or respiratory failure [1]. Preparations should be made for neonatal transfer to a high-acuity neonatal intensive care unit by staff during the delivery [13].

This case highlights important concepts. While these obstetrical skills are taught in residency, the rare frequency of these presentations may lead to increased stress for the ED physician and staff. Preparing for a complicated delivery in the ED should prompt demarcated protocols for managing a high-risk delivery and care for both the mother and neonate. Staff should undergo routine annual competencies to help prepare and familiarize themselves with current practices on the management of malpresentation of the fetus and neonatal resuscitation including the premature infant. The hospital should have methods to elicit immediate help from allied health and/or physicians with delivery and neonatal resuscitation experience.

## Conclusions

In an internal review of the case described above, hospitals without obstetrical services should implement protocols and competencies, particularly in institutions without obstetrical services on site where an imminent delivery would prevent the safe transfer to a receiving hospital with obstetric capabilities. Hospitals should have an emergent delivery code activation to initiate the protocols and summon the appropriate staff for emergent deliveries. ED departments should implement annual reviews and competencies to review and train for high-risk deliveries. These include high-risk delivery simulations, familiarization with obstetric and neonatal equipment in the ED, protocols for delivery and transfer, and maintaining a list of hospital staff with previous obstetrical experience to assist in the delivery. Annual competencies help ED providers maintain current guidelines to manage high-risk delivery and the potential complications in managing a critical mother and infant.
